# P103 A rash decision? A possible capecitabine associated discoid lupus with anti-Ro positivity

**DOI:** 10.1093/rap/rkag113.133

**Published:** 2026-11-03

**Authors:** Abishaa Anandasothy, Shreya Sanjaykumar Kalyani, Darshani PerumpulliArachchige

**Affiliations:** Basingstoke and North Hampshire Hospitals NHS Trust, Basingstoke, United Kingdom; Basingstoke and North Hampshire Hospitals NHS Trust, Basingstoke, United Kingdom; Basingstoke and North Hampshire Hospitals NHS Trust, Basingstoke, United Kingdom

## Abstract

**Introduction:**

Cutaneous lupus erythematosus or discoid lupus can occur in isolation without systemic signs of lupus and are characteristically photosensitive, round, and erythematous patches usually on sun exposed areas. Capecitabine is an oral chemotherapeutic agent that is a prodrug of 5-fluorouracil. It is mainly used in treatment for advanced colorectal, breast and gastric cancer. Common side effects include diarrhoea, vomiting, and palmar-plantar erythrodysesthesia. Although under reported, a case report from France by J Fongue et al reports a very similar case with anti-SSA positivity.

**Case description:**

A 64-year-old woman with stage IV metastatic breast cancer seen by dermatologists for erythematous annular patches, initially appearing on her right forearm and later spreading to her upper arms, chest and neck. On history, it was noted that about five months before the rash, capecitabine was started due to thoracic metastasis whilst on palbociclib and letrozole for five years. A weaning course of steroids and topical steroids were prescribed.

A skin biopsy suggested possible cutaneous subacute/ discoid lupus erythematosus. Notably, there were no significant findings to support drug reaction on the biopsy. However, she was on steroids and capecitabine was paused around the time of the biopsy.

She was referred to the rheumatology service and we discovered a temporal association between the rashes and capecitabine. She reported near complete resolution of the rashes when she was on a treatment break before the current presentation. She did not report any systemic features of a connective tissue disease/vasculitis. She had unremarkable obstetric history.

Her ANA titre was at 1/160 with fine speckled nuclear pattern & fine speckled cytoplasmic pattern. Her ENA profile revealed a positive Anti Ro (SS-A) and Anti Ro-60. Her Anti DsDNA titres and Anti RNP were notably negative. With a CRP of 12 and ESR of 29, which were not far from her baseline for over a decade and largely attributed possibly to an underlying active malignancy, and a normal kidney and liver function, full blood count, clotting profile, and a negative urine dip, she had no other biochemical or serological features of concern from a rheumatological stand point.

Her capecitabine dose was reduced by the oncologists and she remained stable with no new dermatological or systemic features on subsequent reviews by dermatology and rheumatology thus making our impression of a possible diagnoses of capecitabine induced discoid lupus.

**Discussion:**

The mechanism behind capecitabine-induced discoid lupus remains uncertain. There may be a dose related effect as the symptoms improved on dose reduction in our patient.

This case shares several similarities with a previously published report of capecitabine-induced DLE. Dhanju et al. described a patient who developed subacute cutaneous lupus erythematosus approximately 5 months after initiation of capecitabine therapy, with improvement following drug withdrawal. Similarly, in this case, the patient developed DLE after approximately 5 months of treatment, further supporting a delayed autoimmune mediated cutaneous adverse reaction associated with capecitabine exposure. With very few documented cases of capecitabine-induced discoid lupus, this report adds valuable evidence connecting capecitabine to drug-induced DLE.

Clinicians should recognise this adverse effect, as early detection and discontinuation of capecitabine will prevent further complications and avoid unnecessary testing. They should suspect drug-induced DLE in patients with persistent skin eruptions during capecitabine therapy.

This case highlights a rare presentation of capecitabine-induced discoid lupus erythematosus linked to drug exposure and symptom recurrence. Resolution after stopping the drug and flares on restarting indicate capecitabine as the probable cause.

Increased awareness of this uncommon adverse reaction may facilitate earlier diagnosis and management in susceptible patients.

The clear temporal relationship between capecitabine administration and DLE flare-ups underlines the necessity of thorough drug history evaluation when assessing new dermatological and rheumatological symptoms in oncology patients.

Prompt recognition and discontinuation of the offending drug led to substantial improvement, reinforcing the value of early intervention to prevent complications and avoid excessive investigations.

With very few documented cases, this report adds valuable evidence linking capecitabine to drug-induced DLE, supporting the need for increased awareness and reporting of such rare events.

**Key learning points:**

1. To enhance understanding of drug-induced autoimmune dermatological conditions, particularly in oncology settings.

2. To improve diagnostic accuracy and management strategies for rare adverse drug reactions.

3. To discuss multidisciplinary approaches for timely identification and treatment of complex autoimmune presentations.

4. To contribute to the collective knowledge by sharing rare case experiences and fostering collaboration for improved patient outcomes.

